# The impact of artificial intelligence on scientific production: reflections in times of intense technological and social transformation

**DOI:** 10.1590/1984-0462/2026/44/Editorial

**Published:** 2026-07-24

**Authors:** Tulio Konstantyner, Marina Carvalho de Moraes Barros, Fabio Carmona

**Affiliations:** a Universidade Federal de São Paulo, Escola Paulista de Medicina, Departamento de Pediatria, São Paulo, SP, Brazil.; b Universidade de São Paulo, Faculdade de Medicina de Ribeirão Preto, Ribeirão Preto, SP, Brazil

Artificial intelligence (AI) is becoming an increasingly prevalent tool in scientific research, offering significant benefits but also raising important concerns and challenges for authors, reviewers, and journal editors worldwide.^1^

AI offers the potential to combine advantages with practical actions, organized ideas, and textual direction. However, it may also foster an illusion of accuracy, in which the fluency and technical appearance of AI-generated content are mistaken for methodological rigor and validity. Despite its novelty and widespread adoption, there are still few reports on the potential side effects of AI use by researchers, advisors to funding institutions, and editorial committees of scientific journals.^2^

One advantage is the ability to quickly process large volumes of data, enabling complex analyses and identification of patterns that would be hard for a human to detect. AI can also automate repetitive tasks such as literature reviews and text formatting, freeing up time for researchers to focus on more analytical and creative activities. Additionally, advanced AI tools can improve the accuracy of predictive models and experiments.^3^

Reviewers and editors of scientific journals can also benefit from automating initial tasks, such as manuscript screening, plagiarism checking, formatting checks, and identification of basic methodological issues. AI can suggest suitable reviewers based on their areas of expertise. This speeds up the editorial process and reduces the human workload. AI tools can also help to detect manipulated images, statistical inconsistencies, and suspicious references, thereby increasing the integrity of the assessment process.^4^

However, there are disadvantages to using AI that must be considered, which may result from inappropriate use and impaired judgment. One of the main risks is the creation of inaccurate, biased, or fabricated content, particularly when AI systems generate text without rigorous verification. This can compromise scientific reliability. Over-reliance on automated tools can also stunt development and diminish critical thinking, creativity, and autonomy among researchers (this phenomenon has been called AI-induced deskilling). There are also ethical concerns regarding the transparency of algorithms, the use of sensitive data, and the potential for existing biases to be amplified and reinforced, leading to misconceptions that, when repeated often enough, become normalized and accepted as true.^2,5^

AI can generate false positives or negatives when detecting plagiarism, data manipulation, or technical issues, leading to misguided editorial decisions, particularly in busy, high-demand work environments. Another disadvantage is the lack of contextual and interpretive understanding. AI systems may not grasp theoretical or methodological nuances that are clear to human experts. There are also risks associated with over-reliance on these tools, which can impair the reviewers’ critical judgment (deskilling). Ethical issues, such as algorithm transparency and possible built-in biases, can affect the fairness of manuscript evaluation. Reviewers should not upload manuscripts or any confidential material to AI tools, as this may compromise confidentiality and violate editorial policies on data protection and responsible peer review.^6^

It should be noted that AI tools claim neither to ‘measure’ the reliability of the information provided in numbers nor to be certain about anything. Instead, they combine several internal mechanisms to estimate the likelihood of a response being correct.

In simple terms, their reliability is based on four factors:

1. Prior training;
2. Statistical consistency rather than actual factual validation;
3. Security mechanisms such as the clarity of questions and cautious responses to sensitive topics; and
4. Comprehensive searches.

AI does not have direct access to reality or an always-updated database. Therefore, the ‘reliability’ of its actions and the information it provides is an estimate based on training, logical analysis, and, when requested, external verification.^5^

In this context, it is time to reflect on the impact of AI on scientific production, exploring the balance between its advantages (Table 1) and disadvantages (Table 2). As with any new resource, the effects of AI on authors and publishers must be assessed and monitored, as is the case in clinical trials, which assess the efficacy, effectiveness, and safety of a new drug for a specific disease.

**Table 1:** The advantages of using artificial intelligence in scientific production. Proximal, distal, and interactive effects between authors and editors of scientific journals.

Effects*	Researchers	Editors
Proximal	Acceleration of literature reviews (searching for, synthesizing, and comparing studies); Support with scientific writing (improving clarity, cohesion, and standardization); Code generation, preliminary analyses, and simulations; Support with structuring hypotheses, models, and designs; Rapid detection of inconsistencies in manuscripts or data.	Accelerated initial screening (scope, originality, and structure checks); Assistance in evaluating language and ensuring compliance with journal standards; Preliminary detection of plagiarism, redundancy, and AI-generated writing; Support in selecting appropriate reviewers.
Distal	Increased productivity; Democratization of access to scientific writing (fewer language barriers); Greater methodological sophistication, with more advanced modelling tools; Emergence of new lines of research; Reduction in the ‘cognitive cost’ of repetitive science (more time for creativity).	Greater standardization of the initial quality of manuscripts received; Reduction in manual screening and technical evaluation workload; Evolution of editorial criteria to incorporate ethics and transparency in AI usage; Improved volume management and editorial decision-making time; Consolidation of faster, more efficient publishing ecosystems.
Interactive	Quality improvement cycle: Researchers submit clearer texts → editors can process them faster → reviews are more productive → articles are better. Increased competitiveness: More authors produce good texts → editors expand screening technologies → higher standards are demanded. Methodological feedback: The tools adopted by journals (e.g. checkers and semantic evaluators) encourage researchers to adopt the same tools. System integration: Editorial platforms begin to communicate with the writing and analysis software used by authors (e.g. sharing metadata and providing automatic suggestions).	

* Proximal effects: immediate or direct; Distal effects: long-term or indirect; Interaction effects: how one amplifies or alters the other; AI: Artificial Intelligence.

Source: Prepared by the authors.

**Table 2:** The disadvantages of using artificial intelligence in scientific production. Proximal, distal, and interactive effects between authors and editors of scientific journals.

Effects*	Researchers	Editors
Proximal	Risk of excessive reliance on AI for writing, analyzing, or structuring ideas; Generation of superficial, inaccurate, or ‘delusional’ information; Difficulty in maintaining clear and ethical intellectual authorship; Possibility of invisible biases introduced by the model; Misleading sense of conceptual mastery (‘illusion of competence’).	Increase in AI-generated submissions makes screening more difficult; Risk of more sophisticated fraud, such as synthetic data, false citations and manipulated images, is greater; Greater burden of verifying originality and ethics; Disconnect between the appearance of quality, such as polished text, and actual scientific quality.
Distal	Impoverishment of original thinking if AI directs writing and reasoning patterns; Difficulty in differentiating genuine contributions from those generated by tools; Training of researchers who are less competent in essential skills such as writing, argumentation, and critical analysis; Gradual erosion of confidence in authorship and published results.	Growing pressure to detect complex manipulations such as academic deepfakes and fake simulations; Decreased reliability of the peer review process; Possible asymmetries between journals that adopt AI and those that do not; Peer review becomes more expensive, slower, and more technologically complex.
Interactive	Technological escalation: Researchers increasingly use AI, which means that publishers need even stronger AI, creating asymmetric competition between tools. Appearance trap: Manuscripts that are very well written with the help of AI can mask conceptual flaws, which can mislead reviewers and result in weak articles being published. Pressure on integrity: The more AI is used, the harder it is to distinguish "assistance" from "authorship" → ethical and regulatory disputes increase. Ambiguity about merit: The mass adoption of AI can flatten the differences between experienced and inexperienced researchers, making fair evaluation difficult.	

* Proximal effects: immediate or direct; Distal effects: long-term or indirect; Interaction effects: how one amplifies or alters the other. AI: Artificial Intelligence.

Source: Prepared by the authors.

Although AI is a promising tool that surpasses humans in terms of speed, memory, and data processing, human or ‘natural’ intelligence (NI) excels in areas such as consciousness, empathy, deep creativity, ethics, intuition, and meaning. It is important to recognize that AI is developing its capabilities through a dynamic process of interaction with NI. This interaction triggers a process of mutual influence, whereby two intelligences transform over time. NI (users) must carefully understand this phenomenon of co-evolution with reciprocal feedback and be open to beneficial effects (positive feedback), while also creating protective barriers against possible negative effects.^7^ Figure 1 shows the interaction between NI and AI and the necessary actions and precautions during coevolution.

**Figure 1:**
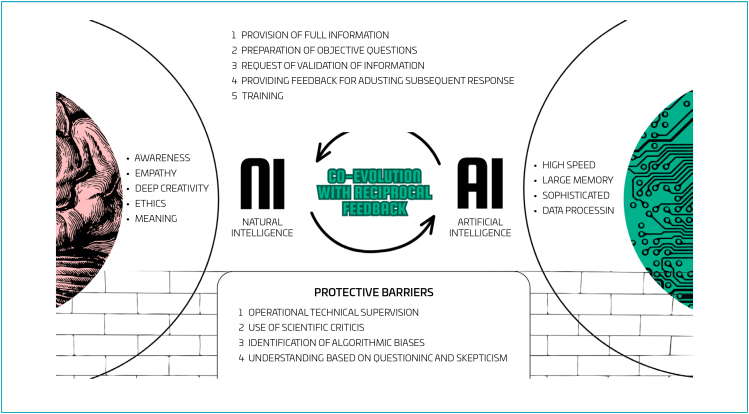
Interaction between natural and artificial intelligence. Co-evolution with reciprocal feedback to build the future: actions and precautions.

Source: Created by the authors.

Although AI offers great opportunities to improve the quality and efficiency of scientific production and can streamline the editorial process, it should always be used critically and in a complementary manner. Therefore, it is unwise to embrace the potential of AI in any scientific context without the oversight of qualified humans. In other words, we should value and incorporate its qualities into our work, combining the available resources thoughtfully and with balance, rather than inadvertently accepting that AI alone determines paths, rules, and truths. Researchers, reviewers, and editors should be familiar with the United Nations Educational, Scientific and Cultural Organization — UNESCO's ethics of AI, which provides internationally agreed principles to guide ethical use, transparency, accountability, and the protection of human rights in scientific practice.^8^

In scientific production, researchers and editors must understand how AI operates and be able to identify algorithmic biases and potential intellectual pitfalls.

When used responsibly, AI can contribute to improving performance through five continuous actions involving interaction between intelligences:

1. Providing information on desired formats and asking clear and objective questions;
2. Providing context or examples to adjust the technical level, style, and focus;
3. Requesting validation of information, such as the confidence level, consistency, and indication of possible errors;
4. Providing useful and sufficient feedback to adjust subsequent responses, and
5. Enabling bilateral and ongoing training.^9^

Figure 1 shows these actions in a reciprocal feedback loop that builds the future.

In line with international recommendations, RPPed is incorporating policies on the use of policies into its guidelines for authors and for reviewers. The general goals of the editorial policy will be, at a minimum, to ensure transparency and accountability related to the use of AI, while also being practical and straightforward.^1,10^

Finally, AI is a powerful tool that can greatly assist researchers and editors in their work, but it cannot replace them. It should not be treated as an absolute authority because it has no consciousness, intentions, or sense of responsibility. It does not think or understand; it only predicts patterns and can make mistakes, ‘hallucinate’ information, or provide convincing but false answers. Ultimately, humans must decide, respond, and be accountable for the consequences.

To use AI appropriately, safely, and critically, researchers require a foundation of technical knowledge, an understanding of ethics, critical thinking skills, and a reflective mindset. Whether the future of scientific production is promising or catastrophic depends on how authors and editors use, value, and, above all, train AI, since they are responsible for the final product.

## Data Availability

There is no database associated with this article. This article did not require Ethics Committee approval.
